# Litter addition decreases plant diversity by suppressing seeding in a semiarid grassland, Northern China

**DOI:** 10.1002/ece3.5532

**Published:** 2019-08-15

**Authors:** Ang Zhang, Dong Wang, Shiqiang Wan

**Affiliations:** ^1^ International Joint Research Laboratory of Global Change Ecology School of Life Sciences Henan University Kaifeng China; ^2^ College of Life Science Hebei University Baoding China

**Keywords:** light interception, seed bank, seed germination, structural equation model, temperate steppe

## Abstract

Plant community diversity is conducive to maintain the regional ecosystems stability and ecosystem services. Seed germination is one of the main ways to regulate plant diversity, owing to seedling recruitment as a basis for plant community renewal. However, the exact mechanism of how plant litter affects seedling recruitment, and species richness is not yet fully understood. Therefore, a litter addition and removal experiment was established in a semiarid grassland to study the effects of plant litter on seedling recruitment and species richness from April to August in 2016 and 2017 in Northern China. The positive correlation between species richness and seedling recruitment indicated that a guarantee of seedling recruitment was the main precondition to protect species richness. Adding rather than removing litter significantly reduced species richness. Litter addition inhibited species richness by directly increasing mechanical damage or indirectly reducing photosynthetically active radiation and seedling recruitment. The results of this study are conducive to understand the evolutionary and regulatory mechanisms of community species richness and seedling recruitment in grassland ecosystems after adding or removing plant litter.

**OPEN RESEARCH BADGES:**

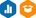
This article has been awarded Open Data, Open Materials and Preregistered research design Badges. All materials and data are publicly accessible via the Open Science Framework at https://doi.org/10.5061/dryad.5dj3jg5 and http://doi.org/10.5061/dryad.13gj03s

## 1 INTRODUCTION

Natural grasslands account for 41% of the earth's surface area and have important ecological functions, such as maintaining biodiversity and stability in terrestrial ecosystems (Firn et al., 2015). Plant community diversity is conducive to maintain the regional resilience, complexity, ecosystems stability, and ecosystem services (Catano & Stout, 2015; Chen, Ma, Xin, Liu, & Wang, 2017). As an important indicator of community diversity, species richness represents the development status of plant communities, has significant feedback to the interference (Liu, Kan, Yang, & Zhang, 2015).

Plant species richness is regulated by complex interactions of related factors (Lamb, 2008). For example, it is not only closely related to productivity (Bai, Han, Wu, Chen, & Li, 2004; McCann, 2000; Williams, Seabloom, Slayback, Stoms, & Viers, 2005), but also to soil properties and ecosystem microclimate (Ives & Carpenter, 2007; Wang, Zhang, Zhu, Yang, & Li, 2018; Wu et al., 2016). As the main target of land use pattern, plant litter is also one of the main factors regulating plant community richness (Foster & Gross, 1998; Muller, Mesléard, Buisson, & Hölzel, 2014; Wardle, Bonner, & Nicholson, 1997). Studies have shown that moderate litter accumulation contributes to improving plant community species richness (Finegan et al., 2015; Nilsson, Xiong, Johansson, & Vought, 1999; Rajaniemi, 2002), while excessive litter accumulation has a negative effect on plant species richness (Kassen, Buckling, Bell, & Rainey, 2000; Lorrillière, Couvet, & Robert, 2012; Tilman et al., 2001). Furthermore, abandonment or closure significant increases the accumulation of plant litter (Hosseinzadeh, Jalilvand, & Tamartash, 2010), which had stimulated effect (Eckstein & Donath, 2005; Rotundo & Aguiar, 2005) or inhibit effect (Jensen et al., 2005; Xiong & Nilsson, 1999) on plant species richness. Mowing and fire, which reduce the amount of plant litter (Davidson et al., 2017; Larreguy, Carrera, & Bertiller, 2017; Trammell, Rhoades, & Bukaveckas, 2004), usually have a significant positive impact on plant community diversity (Ibanez et al., 2018; Wang, Yang, et al., 2018). Therefore, there may be inconsistent responses of plant community species diversity or richness to litter addition and removal.

Plant litter alters plant growth by affecting seed germination, seedling recruitment, and interspecific interactions (Foster, 2001; Xiong & Nilsson, 1999). Moreover, species composition is also regulated by changes in seedling recruitment, which is selected by the litter layer and size of the seed (Egaw & Tsuyuzaki, 2013). However, a few studies have focused on the effect of plant litter on species richness through seedling recruitment. Plant litter plays an important role in seed bank formation and seedling recruitment (Egaw & Tsuyuzaki, 2013). However, plant litter accumulation had significant positive (Bonanomi, Incerti, Antignani, Capodilupo, & Mazzoleni, 2010; Hattenschwiler, Tiunov, & Scheu, 2005) and negative (Foster & Gross, 1998) effects on seedling recruitment, which increased the inconsistency of plant litter effects on plant species richness. In addition, physical conditions (microclimate) indirectly affect species richness by regulating seed germination process and seedling recruitment (Rotundo, Aguiar, & Benech‐Arnold, 2015). For example, improved soil water content stimulates or inhibits seedling recruitment (Dovčiak, Reich, & Frelich, 2003; Ruprecht, Enyedi, Eckstein, & Donath, 2010). Seedling recruitment can be significantly influenced by the accumulated temperature or regulation of day and night temperature (Fennimore, Nyquist, Shaner, Myers, & Foley, 1998; Muhl, Du‐Toit, & Robbertse, 2009). Photosynthetically active radiation (PAR) stimulates or inhibits seedling recruitment and species richness due to plant development, which is regulated by light via the phytochrome pigment (Beligni & Lamattina, 2000; Chory et al., 1996). Therefore, the inconsistent responses of seedling recruitment to physical conditions may result in an uncertain effect of litter on species richness.

A litter manipulation experiment was conducted to explore the responses of plant community seedling recruitment and species richness to a litter addition and removal treatment in a semiarid grassland in Northern China. This field experiment mainly considered two objectives: (a) identify the factors affecting seedling recruitment and species richness of plant communities; (b) determine whether litter addition and removal affects plant species richness through seedling recruitment.

## 2 MATERIALS AND METHODS

### 2.1 Experiment design

This experiment was performed at the Duolun Restoration Ecology Station of the Institute of Botany, Chinese Academy of Sciences (42°02′N, 116°17′E), Inner Mongolia, China. The station is located in a semiarid temperate steppe, at an altitude of 1,324 m. Mean annual precipitation is 385.5 mm, and mean annual temperature is 2.1°C. The sandy soil of the study site is classified as chestnut accord to the Chinese classification, or as Haplic Calcisol accord to the FAO classification (Song, Niu, & Wan, 2016; Xia & Wan, 2013). The plant species at this experimental site are dominated by *Artemisia frigida*, *Agropyron cristatum*, and *Stipa krylovii*. The growing season is from early May to October.

This experiment began in April 2016. A randomized block design was established in our experiment. The natural surface soil (10 cm) was collected and mixed after filtering through a 2 mm sieve to ensure that all plots had the same soil seed bank. The mixed surface soil was placed in 36 ceramic pots of 35 cm diameter to observe seedling recruitment of the plant community in the natural state. The base soil without seed bank was placed at the bottom in each pot. Ten centimeters of mixed surface soil was added on the top of base soil to ensure that the soil seed bank in each pot was uniform. All pots were shallowly buried in the field, where soil was taken. Litter treatments were set at three levels: litter left intact (C), litter removal (LR), and litter addition (LA). Plant litter was collected in the surrounding plot in April 2016 with an average amount of 90 g/m^2^. Plant litter was mixed and added directly to the pot without seed inactivation. The longer litter was cut to 30 cm of debris for easy mixing and adding to the pots. The mixed plant litter (8.66 g) was added to the control plots, and the litter addition plots received 17.32 g of mixed plant litter. There was no plant litter in the litter removal plots. Finally, 5‐cm mesh nylon net was used to cover all plots to fix the litter and eliminate experimental errors. Each treatment was replicated 12 times. This study was implemented during the growing seasons of 2016 and 2017, and the experiment in 2017 was completely repeated the treatments in 2016.

### 2.2 Measurement factors

We set up plots at the end of April of each year and monitored the plots in the subsequent spring and Autumn (April to September). All seedlings were counted and identified to species level until the time of peak emergence, that is, 20 August. The number of seedlings was also counted simultaneously to determine species richness. The census interval was 2–5 days for this experiment. Physical variables were measured simultaneously with seedling recruitment. Soil temperature and moisture were measured in the 5‐cm soil layer. Photosynthetically active radiation was determined at the soil surface in the litter removal plots, but PAR in the litter addition and control plot was determined as the average PAR at canopy height (PAR_c_) and underplant litter (PAR_s_). Light interception (ΔPAR) was defined as the difference between PAR_c_ and PAR_s_.

### 2.3 Statistical analysis

Repeated‐measures analysis of variance (ANOVA) with a random block design was performed to test the main and interactive effects of the plant litter manipulation and year on physical values, seedling recruitment, and species richness. Between‐subject effects were evaluated as a block, by litter manipulation, and their interactions, and the within‐subject effect was year. The effect of block was tested together with the treatment in all analyses, but they were not discussed in this study. One‐way ANOVA and the least significant difference test were used to analyze the differences in the physical values, seedling recruitment, and species richness among litter treatments. A regression analysis was conducted to test the relationship between physical values, seedling recruitment, and species richness. All data were tested for normality and homogeneity of variance prior to ANOVA. All results were analyzed with SAS V8 software (SAS Institute).

The structural equation model (SEM) was used to examine the relationships among physical variables, seedling recruitment, and species richness (Gaitán et al., 2014; Grace, Anderson, Olff, & Scheiner, 2010). The initial model was based on the conceptual model of litter effect on species richness. The species richness and physical values were entered into the SEM. The model was tested using the observed variables to determine whether it was suited for the data. The continuous variables included in the model are described in the initial model setup. As the initial model required the input of successive variables, litter addition or litter remove was entered as a dummy variable (0, 1) in the model. All data were normalized before being entered into the model. The species richness model was divided into the litter addition and removal, respectively, due to the different effects of litter addition and removal on species richness. The chi‐square test of model fit was used to determine whether the fit between the model and data was adequate (*p* > .05). Each path coefficient was divided by its standard error to assess significance. The resulting value followed a *t* distribution, allowing *p* values to be calculated. Given the exploratory nature of these analyses, coefficients with *p* < .1 were considered significant. A thicker line represents a stronger correlation, and the nonsignificant paths were retained as dotted lines in the final model (Lamb, 2008).

## 3 RESULTS

### 3.1 Microclimate

Repeated‐measures ANOVA revealed significant effect of litter manipulation on SM (*F* = 7.09, *p* < .01), ST (*F* = 11.8, *p* < .001), PAR (*F* = 128, *p* < .001), PAR_s_ (*F* = 80.0, *p* < .001), and ∆PAR (*F* = 64.8, *p* < .001). There was a substantial interannual variation of SM (*F* = 3.60, *p* < .05), ST (*F* = 111, *p* < .001), PAR (*F* = 8.70, *p* < .01), PAR_c_ (*F* = 65.1, *p* < .001), and PAR_s_ (*F* = 5.48, *p* < .05) under experimental periods 2 years, but there was no interaction between litter manipulation and year on each microclimate index (Table 1). Litter addition and removal significantly increased and reduced soil moisture by 4.99% and 3.64%, respectively (V/V%, *p* < .05, Table 2). Neither litter addition nor removal affected soil temperature (Table 2). Litter addition significantly inhibited PAR by 11.43% (*p* < .001) via decreasing PAR_c_ by 2.9% (*p* < .001) and PAR_s_ by 25.67% (*p* < .001, Table 2). However, litter removal significantly stimulated PAR by 8.09% (*p* < .001) via increasing PAR_s_ by 11.41% (*p* < .001, Table 2). Litter addition and removal significantly increased and reduced the radiation interception of the ecosystems (ΔPAR) by 78.26% and 33.00%, respectively (*p* < .001, Table 2).

**Table 1:** Effects of litter addition and removal on microclimate, seedling recruitment, and species richness with time

	SM	ST	PAR	PAR_c_	PAR_s_	∆PAR	Seedling recruitment	Species richness
L	7.09**	11.8***	128***	0.66	80.0***	64.8***	25.1***	6.43**
years	3.6*	111***	8.7**	65.1***	5.48*	0.23	9.65**	9.08**
L × years	1.28	2.48^	0.26	0.23	0.55	0.70	1.47	0.21

Note

Linear mixed‐effects model of the effects of the number of treatment years and the number of litter manipulations (litter addition and removal) on microclimate, seedling recruitment, and species richness, with plot nested in year, nested in site, as random effects, using all 30 sites.

Abbreviation: L, litter addition and removal treatments.

Significant level:

^^^ *p* < .1

* *p* < .05

** *p* < .01

*** *p* < .001.

**Table 2:** Effects of litter addition and removal on microclimate: SM, ST, PAR, PAR_c_, PAR_s_, and ΔPAR

Microclimate	LR	C	LA	*F*‐value
SM (%)	18.4 ± 0.25 c	19.1 ± 0.24 b	20.1 ± 0.18 a	13.6***
ST (°C)	25.8 ± 0.49 a	24.9 ± 0.37 ab	24.4 ± 0.38 b	2.88^
PAR (µmol m^−2^ s^−1^)	1,844 ± 12.8 a	1,706 ± 17.8 b	1,512 ± 17.8 c	105***
PAR_c_ (µmol m^−2^ s^−1^)	1,990 ± 11.0 a	1,960 ± 12.5 a	1,903 ± 20.1 b	8.68***
PAR_s_ (µmol m^−2^ s^−1^)	1,700 ± 20.8 a	1,526 ± 39.3 b	1,134 ± 45.8 c	61.8***
ΔPAR (µmol m^−2^ s^−1^)	330 ± 23.6 c	492 ± 38.2 b	877 ± 51.8 a	50.5***

Note

Different letter superscripts indicate a significant difference (*p* < .05).

Abbreviations: C, control; LA, litter addition; LR, litter removal; PAR, Average photosynthetically active radiation; PAR_c_, photosynthetically active radiation in the canopy height; PAR_s_, photosynthetically active radiation in soil surface; SM, soil moisture; ST, soil temperature; ΔPAR, radiation interception.

^^^ *p* < .1

* *p* < .05

** *p* < .01

*** *p* < .001: significant level of *F*‐value.

### 3.2 Effects of litter addition and removal on seedling recruitment and species richness

**Figure 1:**
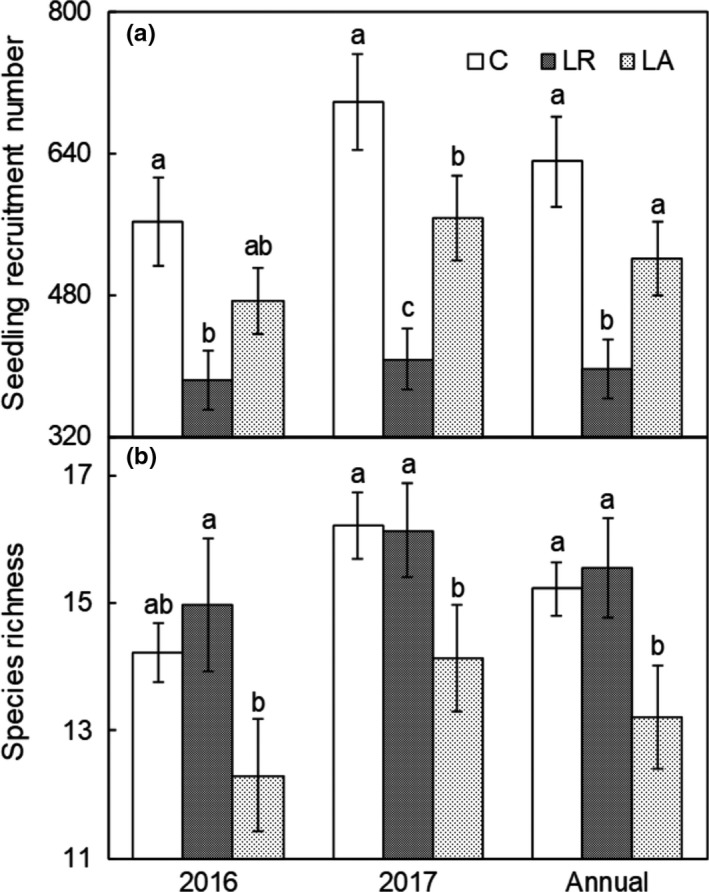
Effects of litter addition and removal on seedling recruitment number and species richness; *n* = 12. C, control; LA, litter addition; LR, litter removal. Error bars indicate the standard error. Different letter superscripts indicate a significant difference (*p* < .05)

Repeated‐measures ANOVA revealed significant effects of litter manipulation on seedling recruitment (*F* = 25.13, *p* < .001). A substantial interannual variation in seedling recruitment was observed (*F* = 9.65, *p* < .01, Table 1) under the litter‐manipulated treatments across the experimental period of 2 years, but there was no interaction between litter manipulation and year on seedling recruitment. Litter removal significantly inhibited seedling recruitment by 37.25% (*p* < .001), whereas litter addition did not affect seedling recruitment, despite that adding litter inhibited seedling recruitment by 17.46% (*p* = .11, Figure 1a and Table 1).

Repeated‐measures ANOVA revealed significant effects of litter manipulation (*F* = 6.43, *p* < .01) on species richness. A substantial interannual variation in species richness was observed under the litter‐manipulated treatments (*F* = 9.08, *p* < .01, Table 1) across the experimental period of 2 years, but no interaction was detected between litter manipulation and year on species richness. Litter addition significantly inhibited species richness by 12.43% (*p* < .05), whereas litter removal did not affect species richness (Figure 1b and Table 1).

### 3.3 Relationship between species richness and seedling recruitment

**Figure 2:**
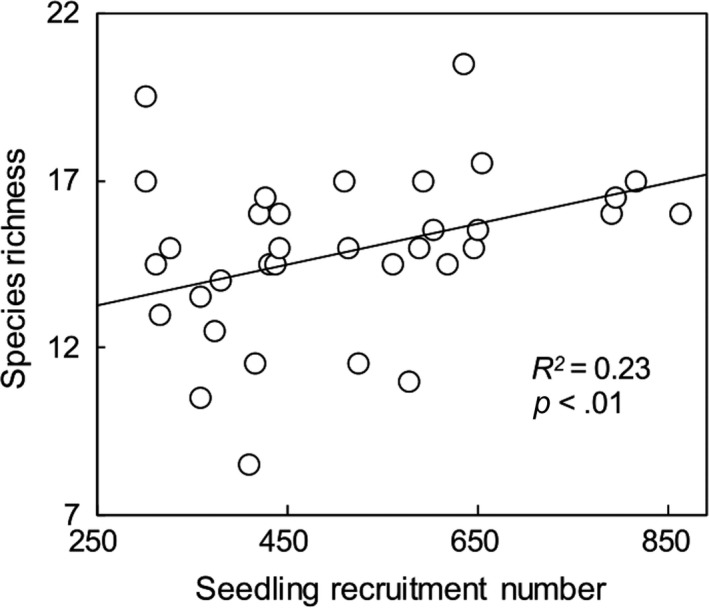
Dependence of the seedling recruitment number on species richness across all 36 plots in the three litter treatments

The annual mean data in all plots were used in the correlation analysis of seedling recruitment and species richness. A significant linear positive correlation was observed between seedling recruitment and species richness (*R* ^2^ = .23, *p* < .01, Figure 2). Furthermore, a significant positive correlation was detected between species richness and seedling recruitment in our SEM, whether it was the litter addition model or litter removal model (Figure 4a,b).

### 3.4 Factors influencing seedling recruitment and species richness

**Figure 3:**
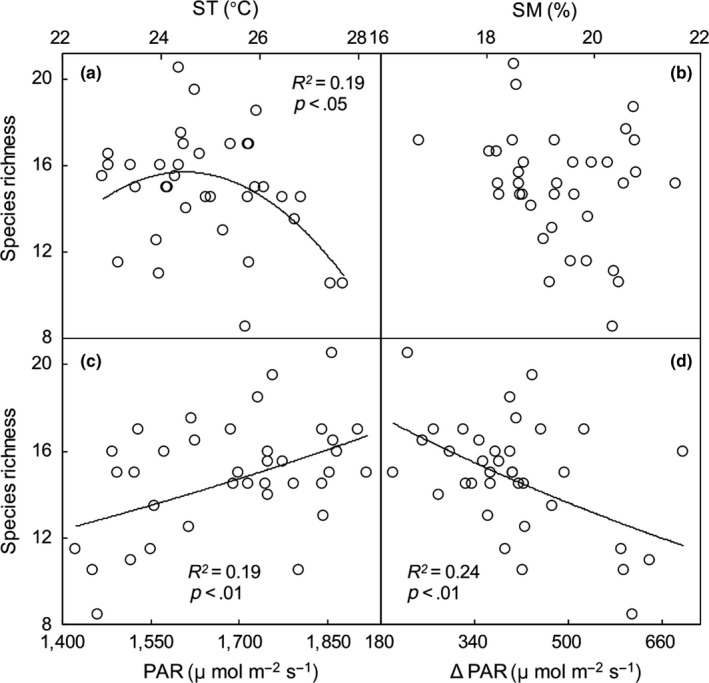
Dependence of soil temperature (ST, a), soil moisture (SM, b), photosynthetically active radiation (PAR, c), and radiation interception (ΔPAR, d) on species richness across all 36 plots in the three litter treatments

The response of species richness to soil temperature was a single peak curve (*R* ^2^ = .19, *p* < .05, Figure 3a). Photosynthetically active radiation was linearly and positively correlated with species richness (*R* ^2^ = .19, *p* < .01, Figure 3c), but soil moisture was not correlated with species richness (Figure 3b). Photosynthetically active radiation and seedling recruitment were the main drivers of species richness. Radiation interception (ΔPAR) was defined as the difference between PAR_c_ and PAR_s_ in this experiment. A significant linear negative correlation was observed between ΔPAR and species richness (*R* ^2^ = .24, *p* < .01, Figure 3d).

### 3.5 Effects of litter addition and removal on seedling recruitment and species richness

**Figure 4:**
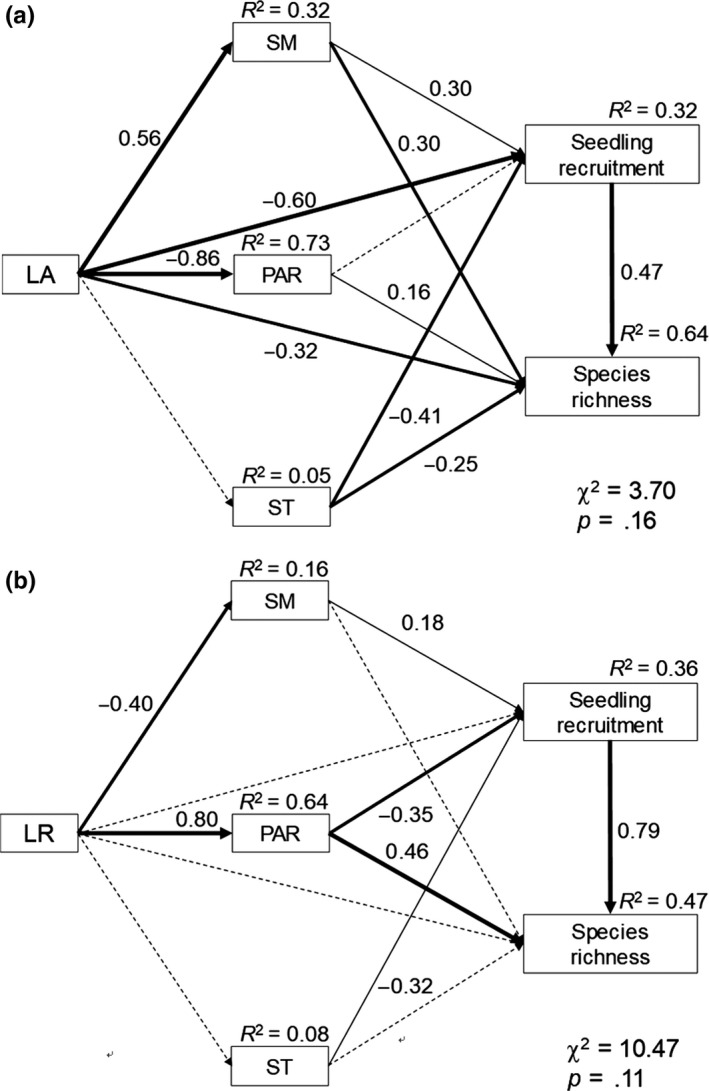
Final structural equation models (SEM) for species richness under the litter addition (a) and litter removal (b) treatments. The thickness of the solid arrows reflects the magnitude of the standardized SEM coefficients. Standardized coefficients are listed beside each significant path

The fit between the species richness model and data was adequate for litter addition (*χ* ^2^ = 3.70, *p* = .16, Figure 4a). We chose to accept this model, as it explained 64.0% of the variation in species richness. Litter addition did not directly affect ST, but it had a direct negative effect and a positive effect on PAR and SM, respectively. Litter addition and ST had a direct negative effect on seedling recruitment, whereas SM had a direct positive effect on seedling recruitment. Litter addition synthetically had a negative effect on seedling recruitment, as the direct negative effect of litter addition transcended the positive effect indirectly by increasing SM. Both SM and PAR had a significant positive effect on species richness, but litter addition and ST directly inhibited species richness. Thus, litter addition inhibiting species richness by significantly and indirectly reducing PAR and seedling recruitment, despite that the increase in SM had an indirectly positive effect on species richness. Moreover, litter addition did not affect seedling recruitment by regulating ST, although ST had a significant positive effect on seedling recruitment in our SEM (Figure 4a).

The fit between the species richness model and data was adequate for litter removal (*χ* ^2^ = 10.47, *p* = .11, Figure 4b). We chose to accept this model because it explained 47.0% of the variation in species richness. Litter removal significantly and directly inhibited and stimulated SM and PAR, respectively, but did not affect ST. Soil temperature and PAR had significant negative effects on seedling recruitment, while SM significantly stimulated seedling recruitment. Litter removal did not directly affect species richness. Litter removal indirectly stimulated species richness by increasing PAR, whereas removing litter indirectly inhibited species richness by decreasing seedling recruitment. However, litter removal did not affect species richness, as the positive effect of increasing PAR offset the negative effect by inhibiting seeding recruitment.

## 4 DISCUSSION

### 4.1 Different regulatory mechanisms of litter addition and removal on seedling recruitment

Litter addition could indirectly affect seedling recruitment by regulating microclimate factors. For example, seedling recruitment occurs immediately after soil moisture increases in summer (Giménez‐benavides, Escudero, & Iriondo, 2007), and adding litter plays a positive role in seedling recruitment by preventing seedling death due to improved soil water content (Patane & Gresta, 2006; Warren, Bahn, & Bradford, 2013). However, soil temperature and radiation were not the major factors regulating seedling recruitment in this study. Furthermore, study has evident that plant litter addition had direct negative effects on seedling recruitment by allelopathy (Ruprecht et al., 2010; Zhang et al., 2015) and mechanical interference (Li & Ma, 2003; Li, Jia, Long, & Zerbe, 2005; Mitschunas, Filser, & Wagner, 2009). These effects may be the main reason for litter addition can directly and significantly inhibit seedling recruitment in this study (Figure 4a). In general, the direct inhibition of litter addition was much greater than that indirect stimulation of increasing water stimulation on seedling recruitment (Figure 4a); Litter addition had a decreasing trend in seedling recruitment (Figure 2a). Litter removal indirectly inhibited seedling recruitment by increasing PAR because strong radiation prolongs seed dormancy as radiation is a stimulus signal that breaks seed dormancy and increases seedling mortality due to loss of water (Aynehb & Afsharinafar, 2012; Jiao, Lau, & Deng, 2007; Valladares et al., 2008). Therefore, removing litter significantly reduces seedling recruitment by stimulating PAR. Moreover, litter removal can indirectly inhibit seedling recruitment by reducing soil moisture (Figure 4b). Moreover, the increase in soil temperature inhibits seedling recruitment by sharply decreasing seed vigor (Auld & Ooi, 2009; Avhad & Marchetti, 2015; Lombraña, Porceddu, Dettori, & Bacchetta, 2016), or accelerating seedlings death at early stage (Binder & Fielder, 1995; Harper & O'Reilly, 2000). Litter removal does not regulate seedling recruitment by affecting ST because ST did not increase in litter removal treatment.

### 4.2 The relationship between seedling recruitment and species richness

Species richness is not only depending on the magnitude and of abiotic factors but also restricted by species regeneration and interactions (Gross, Mittelbach, & Reynolds, 2005). As the basis of plant community richness regeneration, seedling recruitment number is closely related to species richness (Houseman, 2014). Species richness decreased as seedling recruitment increased (Figures 2 and 4a,b), indicating that the number of seedlings recruitment is the main condition regulating species richness. However, litter addition significantly reduced species richness rather than seedling recruitment, whereas litter removal significantly reduced seedling recruitment rather than species richness in our experiment (Figure 1a,b), indicating that manipulating litter may affect the relationship between seedling recruitment and species richness. Plant litter regulates species richness by selectively affecting seedling recruitment of some species. For example, litter inhibits small seeds more than large seeds at the early stage of seedling growth, because seedlings of small species often do not have sufficient energy to break through the soil and litter layer (Paz, Mazer, & Martinez‐Ramos, 2005; Seiwa, Watanabe, Saitoh, Kannu, & Akasaka, 2002). However, a large number of recruit seedlings is an important basis for ensuring species richness, regardless of how litter regulates the seed germination process.

### 4.3 Effects of litter addition and removal on species richness

Previous studies have shown that species richness is inhibited by litter accumulation in high productivity areas (Fang, Xun, Bai, Zhang, & Li, 2012; Nilsson et al., 1999; Sagar, Li, Singh, & Wan, 2019; Su et al., 2018; Xiong & Nilsson, 1999). On the one hand, litter addition inhibits species richness by directly inhibiting seedling recruitment of some species. Litter addition may cause an increase in allelopathy, as some plant litter may inhibit various species by producing a high concentration of toxins (Zhang et al., 2015). On the other hand, litter addition can reduce species richness by maintaining the absolute superiority of dominant species, whose litter prevents recruitment by other species. Therefore, litter addition directly inhibited species richness in our experiments. Furthermore, litter addition indirectly influences species richness by regulating microclimate factors. For example, litter addition indirectly stimulates species richness by increasing soil moisture, as photosynthesis of seedlings is highly and positively correlated with available soil water in an infertile grassland (Davis et al., 1999). Studies have suggested that shading is not conducive to recruitment of seedling populations in communities (Craine & Dybzinski, 2013). The radiation interception of the vegetation canopy and standing litter was the main reason for the significant decrease in species richness (Figure 3e), which further verified that PAR was the main regulator of species richness in the litter‐added plots. The increase in photosynthesis was benefited by the increased PAR, and indirectly increased the growth of underlying or adjacent plants (Davis et al., 1999). Therefore, litter addition indirectly inhibited species richness by reducing PAR.

Litter removal indirectly and significantly stimulated species richness by increasing PAR in our SEM, because increased PAR stimulates species richness by stimulating photosynthesis in most species (Craine & Dybzinski, 2013; Davis et al., 1999), despite some other studies reporting that radiation has no effect on species richness (Dorji, Moe, Klein, & Totland, 2014; Zhou et al., 2019). However, the decrease of SM and the increase of PAR indirectly inhibited species richness by inhibiting seedling recruitment rather than directly affecting species richness in the litter removal plots. Therefore, litter removal has a neutral effect on species richness.

There is a 2‐year short‐term experiment, and the long‐term effects of seedling recruitment and species richness on litter addition and removal need to be further studied. Long‐term overgrazing and mowing reduce seedling recruitment by reducing litter accumulation without affecting diversity. However, long‐term enclosure is harmful to species diversity rather than seedling recruitment owing to litter accumulation. The number of seedlings that colonize is not the only basis for judging the protection of species richness. Soil seed bank, plant phenology, and vegetation growth information should be comprehensively investigated to guide grassland management and biodiversity conservation scientifically.

## 5 CONCLUSIONS

Litter addition inhibited species richness, whereas had no effect on seedling recruitment. Litter removal inhibited seedling recruitment rather than species richness in this study. The number of seedlings that colonize is a major prerequisite for protecting species richness. However, litter addition and removal can regulate species richness by affecting ecosystem resources and microclimate. The negative effect of litter addition on species richness due to the inhibition of allelopathy, mechanical interference, and seedling recruitment exceeded the stimulating effect of soil moisture on species richness. Litter removal did not affect species richness, mainly because the stimulatory effect of photosynthetically active radiation on species richness was offset by the inhibited seedling recruitment on species richness. An enclosure is not conducive to protect species diversity through litter accumulation.

## CONFLICT OF INTEREST

None declared.

## AUTHORS' CONTRIBUTION

Ang Zhang designed the research, collected data, performed the analysis, and wrote the article. All authors contributed critically to the drafts and gave final approval for publication.

## Data Availability

A copy of the data will be archived using the DRYAD international repository (https://doi.org/10.5061/dryad.5dj3jg5).
